# Morphomechanical Alterations Induced by Transforming Growth Factor-β1 in Epithelial Breast Cancer Cells

**DOI:** 10.3390/cancers10070234

**Published:** 2018-07-16

**Authors:** Mariafrancesca Cascione, Valeria De Matteis, Chiara C. Toma, Stefano Leporatti

**Affiliations:** 1 Dipartimento di Scienze Biomediche e Oncologia Umana, Università degli Studi di Bari “Aldo Moro”, p.zza G. Cesare, c/o Policlinico, 70124 Bari, Italy; 2 Dipartimento di Matematica e Fisica “E. De Giorgi”, Università del Salento, Via Monteroni, 73100 Lecce, Italy; valeria.dematteis@unisalento.it (V.D.M.); chiara.toma@unisalento.it (C.C.T.); 3 CNR Nanotec-Istituto di Nanotecnologia, Via Monteroni, c/o Campus Ecotekne, 73100 Lecce, Italy

**Keywords:** TGF-β1, epithelial to mesenchymal transition (EMT), cytomechanics

## Abstract

The Epithelial to mesenchymal transition (EMT) is the process that drives epithelial tumor cells to acquire an invasive phenotype. The role of transforming growth factor-β1 (TGF-β1) in EMT is still debated. We used confocal laser scanning microscopy and scanning force spectroscopy to perform a morphomechanical analysis on epithelial breast cancer cells (MCF-7), comparing them before and after TGF-β1 exogenous stimulation (5 ng/mL for 48 h). After TGF-β1 treatment, loss of cell–cell adherence (mainly due to the reduction of E-cadherin expression of about 24%) and disaggregation of actin cortical fibers were observed in treated MCF-7. In addition, TGF-β1 induced an alteration of MCF-7 nuclei morphology as well as a decrease in the Young’s modulus, owing to a rearrangement that involved the cytoskeletal networks and the nuclear region. These relevant variations in morphological features and mechanical properties, elicited by TGF-β1, suggested an increased capacity of MCF-7 to migrate, which was confirmed by a wound healing assay. By means of our biophysical approach, we highlighted the malignant progression of breast cancer cells induced by TGF-β1 exposure. We are confirming TGF-β1’s role in EMT by means of morphomechanical evidence that could represent a turning point in understanding the molecular mechanisms involved in cancer progression.

## 1. Introduction

The epithelial to mesenchymal transition (EMT) is an essential multi-step morphogenetic process that plays a key role in embryonic development, the differentiation of multiple tissues/organs, and tissue repair; it is also involved in tumor progression [1,2]. When EMT starts, epithelial cells become tumoral, undergoing a critical loss of their typical polarity, which involves both apical and basal membranes. This phenomenon induces the acquisition of a mesenchymal phenotype that makes cells able to infiltrate surrounding tissues [3,4].

The transforming growth factor (TGF)-β cytokines play a key role in the regulation of the growth, development, and homeostasis of tissues [5,6], but their involvement in EMT has also been recognized [7,8,9]. In mammalians, there are three different TGF-β isoforms: among these, -β1 is the most expressed. In extracellular matrix (ECM), the heterodimer TGF-β complex can switch to its active form, and then binds to the type I and II receptors (TβR1 and TβR2) exposed on the plasma membrane. The intracellular domains of TβR1-2 transduce extracellular signals into intracellular responses; through the activation of the Smad complex, they promote the EMT-inducing transcription factors, such as Snail/Slug, ZEB1/2, and Twist [10,11].

Indeed, TGF-β1 induces the loss of E-cadherin expression, triggering the beginning of the EMT program [12,13]. E-cadherin is a transmembrane protein principally involved in homotypic cell adhesion [14], and it is fundamental for sensing biochemical signals deriving from the environment. Owing to its direct link to the cytoskeleton, it plays an important role in specific cellular responses [15,16], concerning internal forces responsible for the epithelial shape and cell movement.

About 50% of carcinomas with extended diffusive characteristics show genetic mutations regarding the locus of E-cadherin [17]. In solid carcinomas, the downregulation of E-cadherin is due to hypermethylation of the respective gene promoters, making it inaccessible to transcription factors and RNA. Instead, a subsequent demethylation of these promoters induces a reactivation of the gene, triggering the reverse process of mesenchymal to epithelial transition [18,19]. Other studies have suggested that the dynamic process of EMT depends also on the E-cadherin/β-catenin complex phosphorylation [20]; in fact, an inappropriate phosphorylation would lead to a breakage of intercellular junctions and a progressive loss of epithelial phenotype. Furthermore, additional endocytosis of E-cadherin increases the migratory ability of tumor cells, decreasing functional cell–cell junctions at the level of the cytoplasmic membrane [9]. Some experimental evidence suggests that these cascades are closely interconnected, so that each one is able to interfere with the others [21]. Taking into account that cytomechanical properties influence shape, motility, and differentiation properties [22,23], we focused on the strict connection between biochemical and mechanical properties (specifically the cell elastic modulus) involved in the carcinogenic transformations. In this context, Atomic Force Microscopy (AFM) is a powerful tool to quantify cell mechanical properties [24]. In addition, confocal laser scanning microscopy (CLSM) permits us to correlate alterations in the elastic modulus with morphological alterations of specific cellular compartments [25]. In this work, we investigated the effects on cytomechanical properties in an epithelial breast cancer cell line (MCF-7) due to the increased TGF-β1 concentration in the cell microenvironment.

## 2. Results

**Figure 1:**
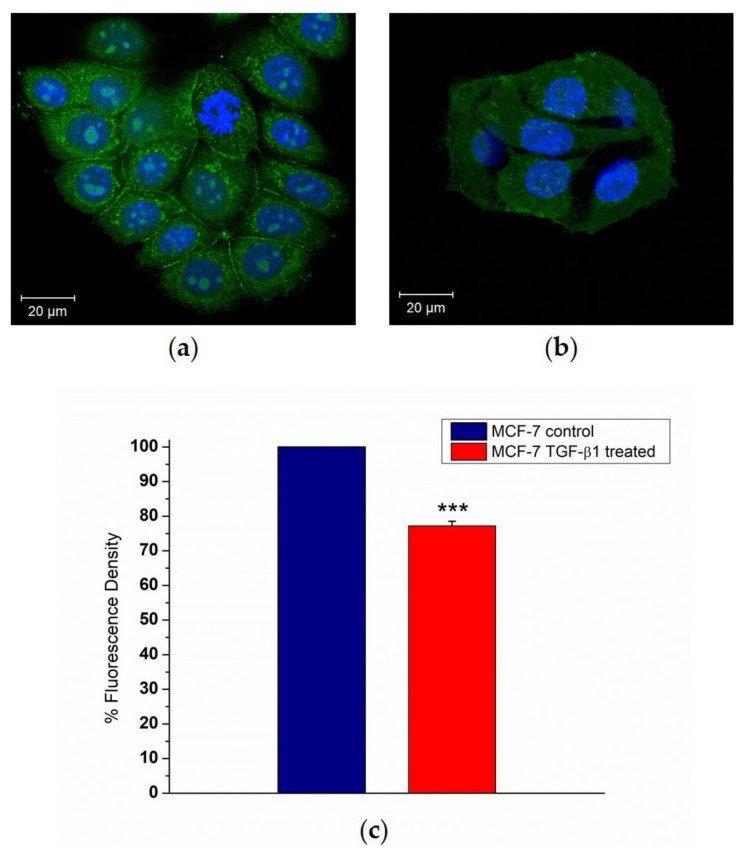
Representative images of immunofluorescence staining of E-cadherin (green) in MCF-7^CTR^ (**a**) and treated with TGF-β1 (5 ng/mL for 48 h), MCF-7^TGF-β1^ cells (**b**). Nuclei are labeled in blue. The images were acquired by Zeiss LSM700 (Zeiss, (Carl Zeiss, Oberkochen, Germany) confocal microscopy. The fluorescence density of E-cadherin (**c**) was quantified by ImageJ specific tool and expressed as a percentage with respect to the control (100%). Results were statistically significant for *p* < 0.05 (<0.05 *, <0.01 **, and <0.005 ***).

The effects of external stimulation of TGF-β1 were evaluated on MCF-7 cells by CLSM in order to understand its potential role in E-cadherin loss. After 48 h of incubation with 5 ng/mL of TGF-β1, confocal images showed appreciable differences in MCF-7^TGF-β1^ compared to the control: as a matter of fact, the treated cells exhibited weaker cell–cell adhesions (Figure 1a,b). These data were confirmed by ImageJ software analysis of E-cadherin-stained images. The fluorescence density parameter was used as a tool to measure the local concentration of this protein (Figure 1c). As shown in the column diagram, the E-cadherin fluorescence density value decreased by about 24% upon external TGF-β1 treatment.

**Figure 2:**
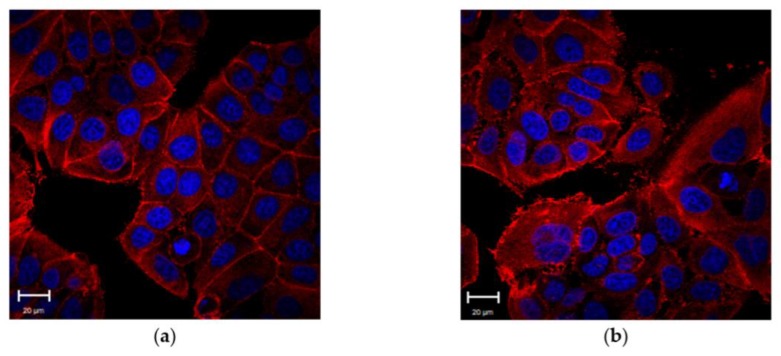
Representative confocal images of control MCF-7 cells (MCF-7^CTR^) (**a**) and treated ones (MCF-7^TGF-β1^). MCF-7^TGF-β1^ were treated with TGF-β1 (5 ng/mL) for 48 h. (**b**). F-actin is labeled in red and nuclei in blue. Acquisitions were performed by Zeiss LSM700 (Zeiss) confocal microscopy.

We also performed confocal analysis of actin fibers to understand whether the TGF-β1 influenced the cytoskeleton physiology of MCF-7 cells. The exposure of TGF-β1 induced a clear reorganization of cellular actin in MCF-7^TGF-β1^ and a distinct pattern in the arrangement of cortical actin between MCF-7^CTR^ and MCF-7^TGF-β1^ was evident. MCF-7^CTR^ exhibited a typical epithelial shape, whereas MCF-7^TGF-β1^ showed a ruffled morphology (Figure 2).

**Figure 3:**
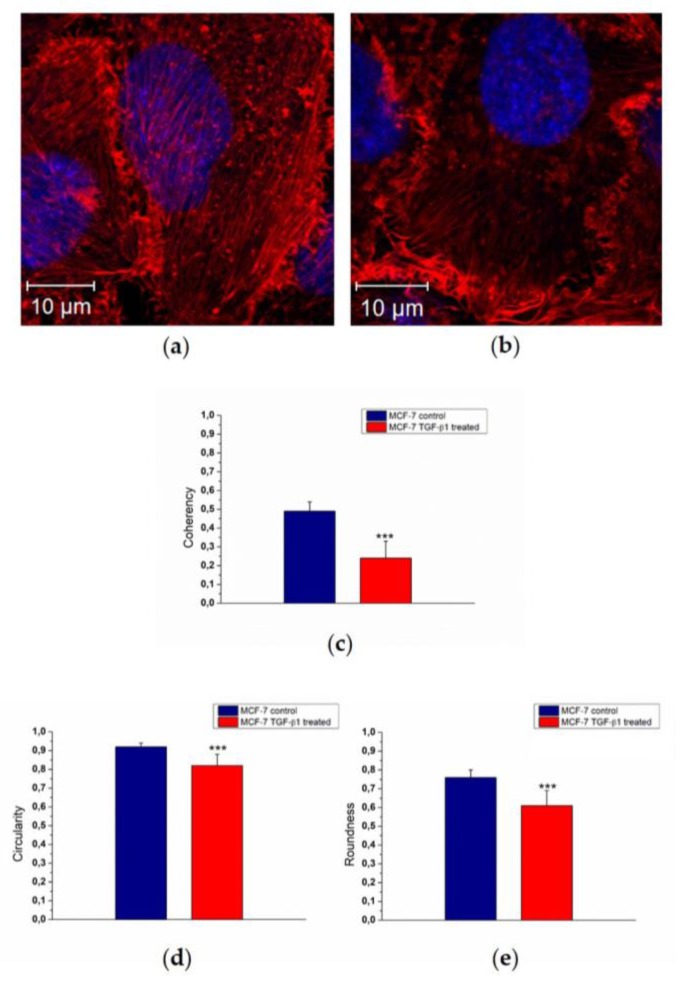
Representative confocal images in apical plane of control MCF-7 cells (MCF-7^CTR^) (**a**) and treated ones (MCF-7^TGF-β1^). MCF-7^TGF-β1^ were treated with TGF-β1 (5 ng/mL) for 48 h. (**b**). F-actin is labeled in red and nuclei in blue. Acquisitions were performed by Zeiss LSM700 (Zeiss) confocal microscopy. In the histograms, the mean values of F-actin coherency (**c**), nuclei circularity (**d**), and roundness (**e**), and their respective standard deviation were reported. Results were statistically significant for *p* < 0.05 (<0.05 *, <0.01 **, and <0.005 ***).

Morphological changes of actin reorganization (Figure 3a,b) were also quantified by means of confocal acquisitions in terms of coherency, as described in our previous work [26] using ImageJ software. Coherency expressed the local orientation of actin fibers: more disordered fibers showed values close to 0, whereas perfectly aligned ones showed a coherency value of about 1. The MCF-7^TGF-β1^ exhibited a decreased coherency value (0.24 ± 0.09) with respect to MCF-7^CTR^ (0.49 ± 0.05) (Figure 3c). To fully understand the experimental evidence regarding the cyto-effects of TGF-β1, we also conducted nuclei analysis alteration in terms of circularity (Figure 3d) and roundness (Figure 3e). The circularity value refers to the presence or absence of surface irregularities on the nucleus surface; the roundness parameter indicates an increasing of its elongated shape.

In MCF-7^TGF-β1^ we found a reduction of both parameters that could indicate a potential migratory behavior of MCF-7^TGF-β1^. In fact, the circularity value changed from (0.92 ± 0.02) to (0.82 ± 0.06) for MCF-7^CTR^ and MCF-7^TGF-β1^, respectively, whereas the roundness values were 0.76 ± 0.04 for MCF-7^CTR^ and 0.61 ± 0.08 for MCF-7^TGF-β1^.

**Figure 4:**
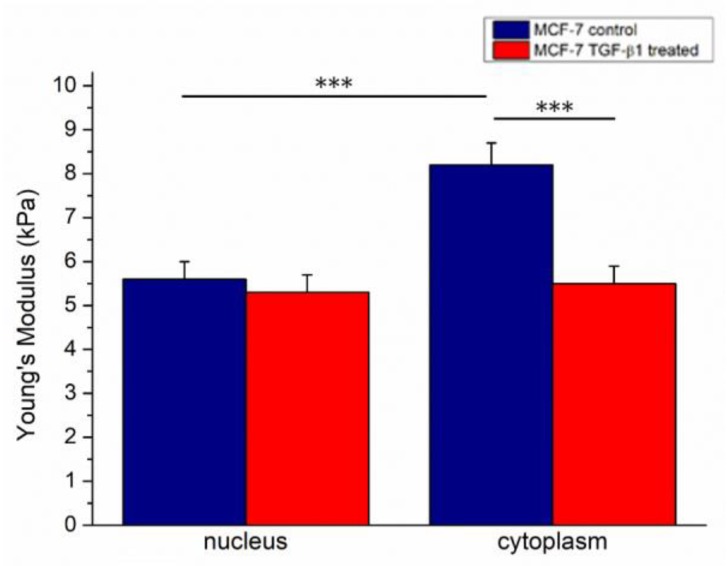
The Young’s modulus values with standard deviation, calculated on 20 cells from the nuclear region and the cytoskeletal area, were reported for MCF-7^CTR^ and MCF-7^TGF-β1^ cells. Results were statistically significant for *p* < 0.05 (<0.05 *, <0.01 **, and <0.005 ***).

The elastic parameter (Young’s modulus, E) of cells was extracted by analysis of force–distance curves, acquired in force volume mode following the procedure described in the Materials and Methods section. Following TGF- β1 treatment, the Young’s modulus values decreased: in MCF-7^CTR^ we found 5.6 ± 0.4 kPa and 8.2 ± 0.5 kPa for the nucleus and cytoplasm, respectively. Conversely, MCF-7^TGF-β1^ showed comparable Young’s modulus values, within the associated measurement error, in the nuclear region and the cytoplasmic one, with E values of (5.3 ± 0.4) kPa and (5.5 ± 0.3) kPa for the nucleus and cytoplasm, respectively (Figure 4).

**Figure 5:**
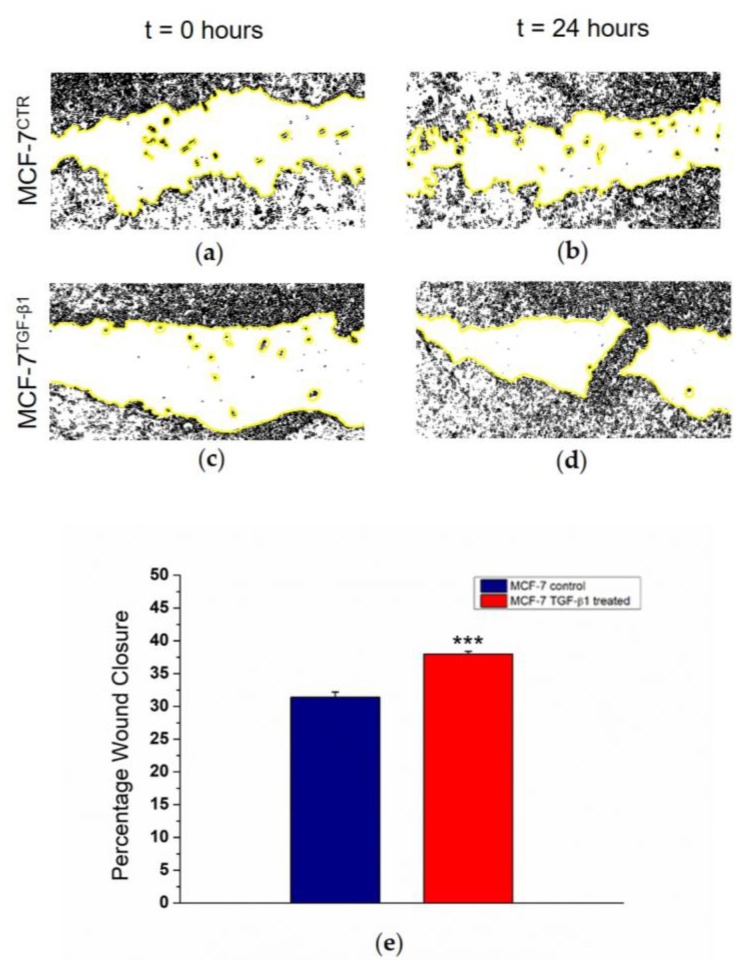
Wound healing assay experiments at two time points (0 and 24 h) on MCF-7^CTR^ (**a**,**b**) and MCF-7^TGF-β1^ (**c**,**d**). The images were acquired in bright field by using a 20× objective (Zeiss) mounted on an Axiovert microscope (Zeiss) and processed by a specific tool of ImageJ software. The yellow lines indicated the boundary of the scratch. The closure percentage (**e**) was calculated as a percentage rate of the area remaining uncovered by the cells (*t* = 24 h) with respect to the area immediately after scratching (*t* = 0).

Finally, a wound healing assay was used because this technique mimics cell migration during wound healing in vivo. We cut MCF-7^CTR^ and MCF-7^TGF-β1^ monolayers and after 24 h we quantified the closure percentage of the scratch, which is related to cell migration over time. We observed an increase in migratory behavior in treated cells (Figure 5a–d), quantified in terms of Percentage of Wound Closure, corresponding to 38% and 32% for MCF-7^TGF-β1^ and for MCF-7^CTR^, respectively (Figure 5e).

## 3. Discussion

The metastatic dissemination of tumor cells is the primary cause of death in patients with cancer [27]. Thus, understanding the molecular mechanisms that underlie tumor progression, local invasion, and formation of tumor metastases represents one of the greatest challenges in exploratory cancer research. Proteomic and genomic studies have demonstrated that cancer cells undergo EMT to promote dissemination from the primary tumor. This program begins with changes in cell–cell and cell–matrix adhesion [28,29,30], involving, among others, TGF-β1 and cytokines, which lead to the activation of several pathways promoting EMT [31]. Many structural alterations take place during EMT, in particular, the loss of cell–cell contacts as well as changes in cell–matrix adhesion, which in turn elicit damage of membrane integrity and consequent alterations in cellular mechanics [32]. In this work, we investigated changes in morphological features and mechanical properties occurring in breast cancer cells upon increased TGF-β1 concentration in the cell microenvironment. In fact, it is widely accepted that the loss of E-cadherin is a hallmark of EMT, correlated with metastatic dissemination in cancer patients [33,34]. For these reasons, we carried out immunofluorescence experiments in order to monitor the reduction of the E-cadherin expression level due to TGF-β1 stimulation, from both a qualitative and a quantitative point of view. Our data were consistent with other experimental evidence reported in the literature [35,36,37]: in fact, the downregulation level of E-cadherin triggered a decrease in cell–cell adhesions and, as a consequence, cytoskeleton remodeling, probably responsible for cellular motility.

Analysis of confocal fluorescence images was performed in order to point out differences in the arrangements of cytoskeletal structures after TGF-β1 treatment and consequent E-cadherin loss. 

MCF-7^CTR^ cells showed an epithelial-like morphology in which neighbor cells were strictly adherent. After TGF-β1 exogenous administration, the cellular pattern appeared less organized. In addition, we observed a loss of cell–cell adherence, irregularities at the edges, and the induction of protrusions at plasma membrane levels. The F-actin filaments in MCF-7^CTR^ were distributed homogeneously on the whole apical surface, whereas in MCF-7^TGF-β1^ actin cortical fibers disaggregated and were mostly localized on the edges of the entire cluster. Furthermore, after treatment, actin fibers exhibited a more isotropic local orientation level in comparison to the untreated sample, as quantified by ImageJ coherency analysis. The TGF-β1 exogenous administration also induced morphological alterations at the nucleus level: in MCF-7^TGF-β1^, they turned out to be more elongated with respect to the control and, on the nuclear membrane, protrusions appeared, as confirmed by ImageJ morphometric analysis (roundness and circularity). As reported in the literature, several factors, involved in tumorigenesis are responsible for the alteration in nuclear shape and margins [38,39]; we speculated that the changed morphology of MCF-7^TGF-β1^ nuclei could suggest a malignant progression.

Moreover, deformation of the nucleus was a consequence of cytoskeletal rearrangement. In detail, the nuclear scaffolds, nucleoli, chromatin, and DNA are linked to the cytoskeletal networks, which are physically coupled to cadherins. For these reasons, TGF-β1 treatment, promoting E-cadherin downregulation, induced structural rearrangements in the cytoplasm and nucleus. Cadherins are associated with β-catenin not only at cell–cell junctions but also in the nucleus; this complex plays an important role in the regulation of gene expression [33]. The E-cadherin downregulation does not affect both pools; β-catenin levels were reduced in the cytosol but no significant differences were observed in the nuclear localization of β-catenin between control and knockdown cells [33]. Keeping this in mind, we believe that the nuclear rearrangement in MCF-7^TGF-β1^ was due to the absence of the E-cadherin/β-catenin complex [40,41].

These observations were confirmed by AFM experiments, performed on living MCF-7 cells, which provided plenty of information about the mechanical properties of MCF-7 after E-cadherin loss, as a direct consequence of TGF-β1 treatment. In this way, samples were in their physiological conditions and we had a good control of many factors affecting the cell mechanics, such as the temperature, cell confluence, and loading rate. In a recent work, Kulkarni et al. [42] demonstrated that MCF-7 cells treated with 5 ng/μL TGF-β exhibited a strong modification of the Young’s modulus value: the authors monitored the cytomechanical behavior at 24 and 48 h, and highlighted that the effects induced by treatment became significant after 48 h. In our work, by using a 1000-fold smaller TGF-β concentration, we found that TGF-β1 exogenous administration influenced elastic behavior of cells: MCF-7^TGF-β1^ cells showed a decreased Young’s modulus compared to the control ones, and treated cells lost their region-specific mechanical properties, mainly in the cytoskeletal region. In addition, the Young’s modulus of the cytoskeletal area in MCF-7^TGF-β1^ turned out to be approximately the same as that of the nucleus. These results suggested that the whole cellular body took part in the mechanical responses, with a rearrangement involving not only the cytoskeletal elements but also the nuclear region, as also previously shown by confocal analysis.

Morphological and mechanical changes in MCF-7 after TGF-β1 exogenous administration suggested an increased capacity of MCF-7 to migrate, which was confirmed by a wound healing assay. Our data highlighted that MCF-7^TGF-β1^ had a more pronounced ability to move from the edges of the scratch onto the free region, restoring the integrity of the monolayer, compared to control counterparts: the augmented migratory capability could represent the starting point of the EMT program. This result is in accordance with biological data presented in the literature, regarding Western blot studies of EMT-related proteins of MCF-7 treated with 5 ng/mL TGF-β1, in which it was demonstrated that the treatment not only induced a reduction of adhesive proteins but also stimulates the expression of mesenchymal markers [36,37]. Thus, our results demonstrated the relationship between E-cadherin loss, induced by TGF-β1 treatment, and biomechanical properties of the breast cancer cells: changes in morphometry, cytoskeletal architecture, elasticity, and migratory capacity suggested the important role of TGF-β1 in the early steps of EMT.

In particular, the elastic parameter represents a critical non-labeled biomarker useful for characterizing cancer cells’ behavior in the metastatic process. From this point of view, scanning force microscopy has proven to be a powerful technique linking biological phenomena and mechanical properties. 

## 4. Materials and Methods

### 4.1. Cell Culture

Human breast cancer cell line MCF-7 was purchased from the American Type Culture Collection (ATCC). Cells were grown in a sterile flask of 25 cm^3^ in Dulbecco’s modified Eagle’s medium (DMEM), supplemented with 10% (*v*/*v*) fetal bovine serum (FBS), 1% (*v*/*v*) L-glutamine, and 1% (*v*/*v*) penicillin/streptomycin, at 37 °C in a humidified atmosphere of 5% CO_2_ (*v*/*v*). All products used for cell culture were purchased from Sigma Aldrich (St. Louis, MO, USA).

### 4.2. Confocal Experiments

MCF-7 cells were seeded at a concentration of 8 × 10^4^ cells/mL and grown until 70–80% confluence in glass Petri dishes (Sarstedt, Germany). Cells were stimulated for a whole 48 h with 5 ng/mL of human recombinant TGF-β1 (Peprotec, Rocky Hill, NJ, USA) and medium replacement using fresh TGF-β1 was done after 24 h (MCF-7^TGF-β1^). MCF-7 cells that were not exposed to TGF-β1 (MCF-7^CTR^) represent the negative control.

The confocal experiments were carried out on fixed (glutaraldehyde at 0.25% in PBS for 10 min) MCF-7^TGF-β1^ and MCF-7^CTR^. After two washes with phosphate-buffered saline (PBS) (Sigma Aldrich), the cells were permeabilized with Triton (Sigma Aldrich) at 0.1% for 5 min and labeled with fluorescent markers: 1μg/mL of phalloidin-TRITC (Sigma Aldrich) for 1 h and 1 μg/mL of Hoechst 33342 (Sigma Aldrich) for 5 min were used to label the actin cytoskeleton and the nuclei, respectively.

The confocal images were acquired by exciting fluorescent proteins—Hoechst and phalloidin-TRITC—with laser radiations having wavelengths of 405 nm and 555 nm, respectively. The Alpha Plan-Apochromat (Zeiss) 100× oil-immersion objective with 1.46 NA was used in the experiments and the acquisition was performed in xy and in z-stack mode. The confocal images were also analyzed on ImageJ 1.47v software (National Institutes of Health, Bethesda, MD, USA) by using specific software tools in order to quantify actin coherency, nuclear roundness, and circularity. Analysis was conducted on 15 cells.

### 4.3. Immunofluorescence Assay

MCF-7^TGF-β1^ and MCF-7^CTR^ were fixed with 4% paraformaldehyde for 15 min and then permeabilized with 0.2% Triton-X100 in PBS for 10 min at room temperature RT. Cells were incubated with a blocking solution (1% BSA in PBST (PBS + 0.1% Tween 20) before the addition of the E-cadherin primary antibody (ab1416, Abcam, Cambridge, UK), diluted 1:50, and used overnight at 4°C. After primary antibody treatment, cells were washed several times with PBS, and afterward samples were incubated for 1 h at RT with Alexa Fluor 488-conjugated anti-mouse secondary antibody (A21200 Thermo Fisher, Waltham, MA, USA), diluted 1:200. Nuclei were stained by Hoechst 33342 (Sigma Aldrich) and immunofluorescence images of MCF-7^TGF-β1^ and MCF-7^CTR^ were acquired with a microscope, under 40× objective. E-cadherin was quantified by ImageJ software through a fluorescence density parameter, which was used as a direct indicator of the local concentration of the protein, previously marked by the antibodies.

As reported in the ImageJ User guide, integrated density corresponds to the sum of value pixels in a specific image selection. The fluorescent RGB acquisition was converted to a grayscale image, and, after calibration, the integrated density was calculated as (Selected Area) × (Mean Gray Value). In our case, fluorescence density on E-cadherin was a direct indicator of the local amount of the protein. It was measured by selecting the whole cell area of 15 MCF-7TGF-β1 cells and 15 MCF-7CTR cells, respectively. The fluorescence density mean values obtained for MCF-7^TGF-β1^ cells and MCF-7^CTR^ cells were expressed as a percentage with respect to the control (MCF-7^CTR^ 100%), allowing us to quantify the relative amount of E-cadherin in MCF-7^TGF-β1^ cells compared to control ones.

### 4.4. AFM Experiments

MCF-7 cells were seeded in plastic Petri dishes (Corning, New York, NY, USA)) at 8 × 10^4^ cells/mL concentration. When 70–80% confluence was reached, they were stimulated for a whole 48 h with 5 ng/mL of human recombinant TGF-β1 (Peprotec) and medium replacement using fresh TGF-β1 was done after 24 h. Immediately before performing AFM measurements, the MCF-7^CTR^ and MCF-7^TGF-β1^ cells were washed with sterile PBS solution and the medium was replaced with 3 mL of Lebovitz (L-15) medium (Sigma Aldrich).

The elastic behavior of living MCF-7^CTR^ MCF-7^TGF-β1^ cells was extracted by AFM measurements conducted in force volume mode (FV), using the following acquisition parameters: Scan area 50 μm, Ramp rate 4.88 Hz, FV scan rate 0.03 Hz, Trigger Threshold 50 nm, Number of sample 512, Sample per line 128, Lines 128.

The force volume experiments were performed on i = 15 MCF-7^CTR^ and i = 15 MCF-7^TGF-β1^ cells. From each cell, 50 force–distance curves were exported: j = 25 curves were extracted in correspondence of nuclear region and j = 25 in cytoplasmic area too. The local Young’s modulus *E* was evaluated by fitting the experimental loading data (height *z*, cantilever deflection *δ_c_*) with a modified Sneddon model:

(1) $$-k_{c}\delta_{c}=\frac{2Etg\theta }{\pi \left(1-\nu^{2}\right)}{\left(z-\delta_{c}\right)}^{2},$$

where the Poisson ratio ν was fixed to 0.5 (typical value for living cell [43]). *θ* is the half-angle of the conical tip and *k_c_* is the elastic constant value of the cantilever; these parameters depend on the AFM probe used. Our experiments were carried out using a V-shaped Bruker’s Sharp Microlever (MSNL, tip C), which consists of a high-sensitivity Silicon Nitride cantilever having a nominal elastic constant of 0.01 N/m; prior to carrying out each measurement, this value was accurately estimated by the thermal tune method [44].

The analysis was conducted using Nanoscope Analysis software (Bruker Inc., Billerica, MA, USA); the employed algorithm treated the contact point as the fit variable and considered the adhesion forces. For each cell, the Young’s modulus values (E_nucleus_ and E_cytoplasm_) were calculated as the average of j = 25 values obtained from each extracted curve and its associated errors as the standard deviation. Then, for MCF-7^CTR^ and MCF-7^TGF-β1^, the E values were calculated as the weighted average on E_nucleus_^i^ and E_cytoplasm_^i^ values and the associated error was estimated using the theory of error propagation. The elasticity data were analyzed and graphed using the OriginPro software (OriginLab version 8, Northampton, MA, USA) A two-tailed *t* test was used to test the statistical significance of the results: in detail, the values of Young’s modulus corresponding to the two cellular regions of each sample were compared, and the differences were considered statistically significant for *p*-values < 0.05.

### 4.5. Wound Healing Assay

MCF-7^TGF-β1^ and MCF-7^CTR^ monolayers were wounded with a plastic 200-μL pipette tip, washed with medium to remove detached cells, and then maintained in standard culture conditions for 24 h. In order to evaluate wound healing, images of the cells were acquired immediately afterward the scratch (*t* = 0) and after 24 h, in a bright field by using a 20× objective (Zeiss) mounted on an Axiovert microscope (Zeiss). Cell migration was assayed by the measurement of wound closure in the scratch region, using the ImageJ public domain software (NIH). In detail, the cell-free area in the captured images was manually traced and quantified by ImageJ specific tools; after that the closure percentage was calculated, as reported in [45,46], as:

(2) $$Wound\;Closure\;Percentage=\;\left(\frac{A_{0}-A_{t}}{A_{0}}\right)\times 100\%,$$

where *A*_0_ and *A_t_* represent the area immediately after scratching and the area of the scratch after 24 h, respectively.

## 5. Conclusions

Nowadays, the rise of nanotechnologies has spurred the development of complementary approaches to understand tumoral progression. The scanning force investigation provides new insight into EMT concerning the role of TGF-β; in fact, knowledge of the biomechanical behavior along with the biological evidence permits a full characterization of the disease state at a cellular level.

In our study, the actin reorganization and nuclei remodeling were correlated to altered Young’s modulus and migration capabilities in breast cancer cells exogenously stimulated with TGF-β1. We demonstrated that TGF-β1 promotes a rearrangement of cell structures, which corresponds to an increased elasticity and suggests an increased cell migratory capability.

Our biophysical approach opens up a wide range of applications in the functional analysis of nanomolecular complexes in living cells, highlighting their relevance to the onset and development of the disease. In fact, the investigation of the morphomechanical properties of cells could represent a turning point in understanding the mechanisms involved in cancer progression. This new approach will be fundamental to the future design of pharmacological strategies and, as a consequence, will help prevent breast cancer complications related to metastasis.
